# Firearm Screening and Counseling in General Medicine Primary Care Clinics at an Academic Medical Center

**DOI:** 10.1007/s11606-023-08379-x

**Published:** 2023-08-31

**Authors:** Joseph Ladines-Lim, Kayla Secrest, Autumn Pu, Aaron Sifuentes, Elizabeth Spranger, Jennifer Stojan, Jennifer Meddings

**Affiliations:** 1 Department of Internal Medicine, University of Michigan, Michigan Medicine, Ann Arbor, MI USA; 2 Department of Pediatrics, University of Michigan, Michigan Medicine, 3116 Taubman Center, SPC5368, 1500 East Medical Center Drive, Ann Arbor, MI 48109 USA; 3 Center for Clinical Management Research, Veterans Affairs (VA) Ann Arbor Healthcare System, Ann Arbor, MI USA

## INTRODUCTION

The American College of Physicians (ACP) advocates that physicians perform firearm screening and safety counseling (e.g., best storage practices),^1^ for which there exists relevant guidance.^2, 3^ However, it remains unclear how often internal medicine (IM) providers perform this and perceive this recommendation.^4^ At our institution, we surveyed IM outpatient providers and retrospectively reviewed charts for new patients’ health maintenance exam (HME) documentation, including routine pre-visit questionnaires containing firearm screening.

## METHODS

Our institution is a large, suburban academic tertiary center that serves Michigan and much of the surrounding region. In June 2022, as part of an IM resident-driven quality improvement project, we surveyed IM residents and primary care faculty through convenience sampling regarding firearm safety counseling, including prior training; frequency viewing and addressing patient responses to the firearm access pre-visit screening question, provided to all new patients; and comfort level and sense of responsibility regarding the issue. Notably, no curriculum on firearm safety for internists existed at our institution at the time of the survey.

We also retrospectively reviewed charts of all new HME visits in May 2022 at all IM primary care clinics, using a standardized abstraction form to assess the following: firearm access screening pre-visit responses provided by patients (paper or online); history of psychiatric or substance use disorder (SUD) given the ACP’s position that this be included in background checks for anyone purchasing firearms;^1^ and any documentation of counseling.

The University of Michigan Medical School Institutional Review Board assessed this study as non-research and waived ethics approval requirements.

## RESULTS

Representing 10 clinic sites, 109 of 226 providers (43% faculty, 57% residents) completed the survey (48% response rate). Among providers, 32% were unaware of the pre-visit screening question and 89% had no related training. Self-reported practice, comfort, and perceived importance of the issue were variable (Table 1). During HME visits, 61% sometimes or never address firearm safety; only 36% felt comfortable doing so. The issue was considered at least very important to address by 39%, while 32% felt it was slightly or not at all important. Although 45% agreed firearm safety falls within their role, 33% disagreed. Most felt more likely to address the issue in patients with mental illness and SUD. Barriers to addressing firearm safety included lack of training and time constraints.

**Table 1:** Provider Survey Responses Regarding Firearm Screening and Related Training

Survey questions	Provider responses*							
	No			Yes				
Are you aware of the question that screens for firearm access in new patient visits? This question asks, “Do you have a gun at home?” (*N* = 109)	31.5 ± 6.1			68.5 ± 6.1				
Have you received any training in addressing firearm access and safety at home for patients in clinic? (*N* = 107)	89.2 ± 4.2			10.8 ± 4.2				
	Never	Sometimes	About half the time			Most of the time		Always
How often do you view the response to the questions screening for firearm access at home? (*N* = 108)	27.0 ± 5.9	26.1 ± 5.8	7.2 ± 3.4			28.8 ± 6.0		9.9 ± 4.0
How often do you address the issue of firearm access with your patients if they screen positively? (*N* = 107)	30.6 ± 6.2	31.5 ± 6.2	8.1 ± 3.6			16.2 ± 4.9		11.7 ± 4.3
How often do you document any counseling provider on firearm access? (*N* = 109)	46.8 ± 6.5	39.6 ± 6.4	0.9 ± 1.2			7.2 ± 3.4		5.4 ± 3.0
Survey questions	Provider responses*							
	Extremely uncomfortable	Somewhat uncomfortable	Neither comfortable nor uncomfortable		Somewhat comfortable		Extremely comfortable	
How comfortable do you feel providing anticipatory guidance/counseling to a patient who screens positive for firearm access at home? (*N* = 109)	9.0 ± 3.8	26.1 ± 5.8	29.7 ± 6.0		28.8 ± 5.9		6.3 ± 3.2	
	Not at all important	Slightly important	Moderately important		Very important		Extremely important	
How important do you think screening for firearm access at home is in an initial new patient visit? (*N* = 109)	3.6 ± 2.4	28.8 ± 5.9	27.9 ± 5.9		24.3 ± 5.6		15.3 ± 4.7	
	Strongly disagree	Somewhat disagree	Neither agree nor disagree		Somewhat agree		Strongly agree	
How much do you agree with the following statement? “A primary care provider is responsible for addressing firearm access and safety in a new patient appointment.” (*N* = 109)	8.1 ± 3.6	25.2 ± 5.7	21.6 ± 5.4		36.0 ± 6.3		9.0 ± 3.8	
	Extremely unlikely	Unlikely	Neutral		Likely		Extremely likely	
How much more likely are you to address the issue of firearm access at home in a patient with history of mental illness? (*N* = 109)	0.0 ± 0.0	1.8 ± 1.7	20.7 ± 5.3		51.4 ± 6.6		26.1 ± 5.8	
How much more likely are you to address the issue of firearm access at home in a patient with history of substance abuse? (*N* = 109)	0.9 ± 1.2	4.5 ± 2.7	45.0 ± 6.5		37.8 ± 6.4		11.7 ± 4.2	

Provider survey responses regarding firearm screening and related training, including frequency with which providers address firearm access in clinic if patients screen positively; comfort level with providing anticipatory guidance/counseling to patients who screen positively; perceived importance of screening for firearm access in a new patient visit; and feeling of agreement or disagreement with the statement that “A primary care provider is responsible for addressing firearm access and safety in a new patient appointment”

^*^Responses are reported as percentages of the reported sample size *N* ± 95% confidence interval. Percentages may not add up to 100% due to rounding

We reviewed 501 charts. Only 44% of patients answered the screening question, with 26% of these 44% reporting firearms at home. Notably, 96% of patients who skipped the firearm question still completed the remaining questionnaire. Of those with firearms, 30% and 9% had psychiatric and SUD history, respectively. No charts had any documentation of counseling.

## DISCUSSION

There is evidence firearm safety counseling can result in safer storage practices.^5^ With a reasonable survey response rate, our study at an academic center demonstrated important findings regarding screening and counseling, including lack of provider comfort doing so, low perceived responsibility, and no documentation of counseling. Despite persistent calls for action from professional organizations, our study showed no improvements in feelings of responsibility or rates of counseling compared to a 2014 provider survey that found that over half of ACP members agreed physicians should be involved in firearm injury prevention.^4^ Another recent survey of residents at another academic institution similarly showed lack of comfort with the issue due to lack of training and barriers such as time constraints, though they also demonstrated high interest in related training.^6^

Additionally, our study delves beyond prior studies’ scope through chart review, revealing most strikingly that of the 56% of patients who did not answer the firearm access screening question, the vast majority (96% of the 56%) did answer all other pre-visit screening questions, suggesting the decision to not answer was deliberate. This, coupled with survey responses, suggests generalized resistance from patients and providers alike to discuss firearm safety, though it is worth noting again that IM providers at our institution at the time of our study had no formal training available, a key barrier cited in our survey. There is also evidence that patients are not completely resistant to conversations around firearm ownership, though the discordance of our own findings underscores the issue’s complexity.^7^ Qualitative methods may be warranted to better understand apparent resistance to engagement and inform appropriate strategies for firearm screening and counseling.

## Data Availability

Data for this study is available upon request.

## Abbreviations

ACP: American College of Physicians
IM: Internal medicine
HME: Health maintenance exam
SUD: Substance use disorder
